# *Ganoderma lucidum* cultivation affect microbial community structure of soil, wood segments and tree roots

**DOI:** 10.1038/s41598-020-60362-2

**Published:** 2020-02-26

**Authors:** Fei Ren, Yuguang Zhang, Hai Yu, Yong An Zhang

**Affiliations:** 1 Forestry experiment center in north China, Chinese Academy of Forestry, Beijing, China; 2 Research Institute of Forest Ecology, Environment and Protection, Chinese Academy of Forestry, Beijing, China; 3 Institute of Cereal & Oil Science and Technology, Academy of National Food and Strategic Reserves Administration, Beijing, China

**Keywords:** Ecology, Microbiology

## Abstract

The popular medicinal mushroom *Ganoderma lucidum* (Fr.) Karst. [Ling Zhi] has been widely used for the general promotion of health and longevity in Asian countries. Continuous cultivation may affect soil microbe and soil properties. However, the effect of *G. lucidum* cultivation on related wood segments, soil and tree roots microbial communities and soil properties is remain unknown. In our study, the microbial communities of soils, wood segments, and tree roots before and after *G. lucidum* cultivation were investigated by Illumina Miseq sequencing of both ITS and 16S rDNA, and taxonomic composition of eukaryotic and prokaryotic microorganisms were observed. Indices of microbial richness, diversity and evenness significantly differed between before and after *G. lucidum* cultivation. Each of the investigated sampling type harbored a distinctive microbial community and differed remarkably before and after *G. lucidum* cultivation. Ascomycota and Basidiomycota (fungi), Proteobacteria and Actinobacteria (bacteria) showed significant differences after Ling Zhi cultivation. The soil property values also changed after cultivation. The redundancy analysis (RDA) showed that both the fungal and bacterial community structure significantly correlated with soil humus, pH, nitrogen, carbon and trace elements (Fe, Zn, Mn, Cu) contents. The results indicated that *G. lucidum* cultivation may have significant differed the associated microbial community structures and soil properties. The study will provide useful information for *G. lucidum* cultivation and under-forest economic development.

## Introduction

*Ganoderma lucidum* (Ling Zhi or reishi mushroom,) has been an economically important mushroom, especially in the East Asian countries (i.e. China, Japan, Korea), since 4000 years ago^1^. The fungi is grown on a widespread commercial scale and is commonly consumed for medicinal and spiritual potency^1–3^. Ling Zhi cultivation under forests has great economic and ecological importance. Problems such as production ability limit, affecting forest resources etc. exist^4^. Cultivation (continuous monocropping) can lead to disease infection, microbial community composition change, alterations in soil quality and plant growth^5–8^. Similarly, Ling Zhi cultivation may play a vital role on microbial communities of soils, wood segments and tree roots as well as soil properties. Meanwhile, related microbial communities could also affect soil quality and Ling Zhi cultivation. However, information on effect of *G. lucidum* cultivation on these microbial communities and soil properties is still unavailable, making it very difficult to draw any general conclusions.

For microbial community researches, culture-dependent and culture-independent approaches can be adopted^9,10^. Currently, high-throughput sequencing provides a deep understanding of the biotic and abiotic parameters affecting microbial community composition, having great advantage of generating huge data as well as detecting unculturable species at much lower price, making it easier to extend our knowledge of microbial diversity^11–13^.

Our main study aim is to investigate the differences on the microbial communities of soils, wood segments and tree roots before and after *G. lucidum* cultivation. Correlation between the microbial community structure and the soil properties is also elucidated. The study will provide a comprehensive view of microbial community and soil properties changes affected by *G. lucidum* cultivation and useful information for further exploitation and utilization.

## Materials and Methods

### Ethics

The samples in the study were collected on private land and the owners allowed the full permission to conduct the study on the sites. The experimental materials did not involve in any humans and animals.

### Sample collection

The study was carried out in the sampling sites (ca. 100 m × 100 m) of Jiu Long Mountain Forestry Reserve area (39°56′15″N, 116°05′12″E), Mentougou District, Beijing, North China. The samples were collected twice: 1) before *G. lucidum* cultivation—in October 2015; 2) three years after *G. lucidum* cultivation—in October 2018. Apart from *G. lucidum* cultivation, all the other conditions were same. Under forests, Ling Zhi grows on the wood segments, wood segments are half in the soils under forests trees *Pinus tabulaeformis* Carr. (Supplementary Fig. S1). Each time, three types of samples were collected: soil, wood segments where *G. lucidum* directly grow and *P. tabulaeformis* roots in the site. Four biological replicates were chosen for each sample. The sampling sites were evenly divided into four plots (each ca. 50 m $\times$ 50 m). For soil samples, nine soil cores (2.5 cm diameter) from 0 to 30 cm depth (where directly in touch with wood segments) were randomly collected and mixed for each plot. For wood segments and tree roots, also, nine were randomly collected and mixed for each plot.Non-cultivated negative control samples were set up to make sure the effect of Ling zhi cultivation. Samples were collected into sterile plastic bags and processed within 24 h. Soil physicochemical properties (soil carbon, nitrogen, phosphorus, potassium, sodium, humus, copper, iron, manganese, zinc, pH) were tested at Pony Testing International Group Ltd (Beijing) with standard method in^14^.

### DNA extraction, amplification of ITS and 16 rDNA region and Illumina sequencing

TIANamp Soil DNA Kit (TIANGEN Biotech Co., Ltd., Beijing, China) was used to extract DNA from homogenized soil samples. Genomic DNA of the wood segments and tree roots were extracted with a standard cetyl–trimethyl ammonium bromide (CTAB) method with modifications^15^. The concentrations of the DNA were measured with NanoDrop ND-2000 spectrophotometer (Thermo Fisher Scientific, USA).

Fungal primers ITS1F (CTTGGTCATTTAGAGGAAGTAA) and ITS2R (GCTGCGTTCTTCATC GATGC) were used to amplify ITS1 region of rDNA^16^. Bacterial 16S rDNA was amplified with primers 338 F (ACTCCTACGGGAGGCAGCA) and 806 R (GGACTACHVGGGTWTCTAAT)^17^. The PCR products were purified and sequenced with Illumina MiSeq. platform at Shanghai Majorbio Science and Technology Ltd. All sequences were deposited at the Sequence Read Archive (SRA) of the National Center for Biotechnology Information (NCBI) under project accession number PRJNA538936.

### Pre-processing and analysis of ITS rDNA sequences

Raw reads quality was pre-processed with FLASH^18^ and Trimmomatic^19^. Mothur standard operation pipeline (SOP, v.1.37.6)^20^ was used to analyze the data and classify sequences into OTUs (Operational Taxonomic Units) at 97% similarity against UNITE Database v. 7.2^21^ (fungi) and Silva Database release 128^22^ (bacteria). Sequence reads were subsampled for each sample with the minimum number (43410 fungi; 41074 bacteria) of reads among all samples before comparative analysis. The indices richness (Sobs), diversity (Invsimpson) and evenness (Simpsoneven)^23^ were calculated in Mothur. Data for rarefaction curves were also generated in Mothur. One-way ANOVA (analysis of variance) was used to identify differences in community richness, diversity and evenness, and microbial abundance. R language platform^24^ was used for analysis and visualization of the data sets of the microbial diversity and abundances in different samples (Rarefaction curves, Venn, bar chart, PCoA, RDA, PERMANOVA).

## Results

### MiSeq sequencing data

In the study, after sequence quality evaluation, a total of 1,305,273 fungal and 1,217,144 bacterial high quality sequences were recovered. The samples were with an average of 54,386 ± 2952 fungal and 50,714 ± 1348 bacterial sequences (mean ± SD). The average sequence length was 272 bp and 438 bp for fungi and bacteria, respectively.

### Richness, diversity and evenness of microbial communities

Quality-filtered sequences were clustered into 1,831 fungal OTUs and 3,549 bacterial OTUs (excluding singletons). The soil samples had the highest richness, diversity and evenness, and the wood segments had the lowest richness, diversity and evenness in all the microbial communities (Table 1). The fungi showed higher richness, diversity and evenness in soil and wood segments samples after *G. lucidum* cultivation. The bacteria indexes are higher in wood segments after cultivation, while, showed an opposite trend in soil and tree root samples (Table 1). Microbial richness, diversity and evenness indexes of samples showing statistically significant differences were shown in Supplementary Fig. S2 labeled with asterisk (*). Rarefaction curve showed that the OTUs abundance were saturated in all samples (Supplementary Fig. S3A,B). [LA– Soil after *G. lucidum* cultivation; LB– Soil before *G. lucidum* cultivation; MA–Wood segments after *G. lucidum* cultivation; MB–Wood segments before *G. lucidum* cultivation; GA– *P. tabulaeformis* roots of the sites after *G. lucidum* cultivation; GB– *P. tabulaeformis* roots of the sites before *G. lucidum* cultivation].

**Table 1:** Richness, diversity and evenness indexes of microbial communities (mean ± SD).

Samples	Microbe	Sobs (richness)	Invsimpson (diversity)	Simpsoneven (evenness)
LA	Fungi	667.00 ± 5.39	26.54 ± 0.74	0.040 ± 0.004
	Bacteria	2542.757 ± 30.37	94.617 ± 2.75	0.038 ± 0.005
LB	Fungi	413.75 ± 31.80	12.85 ± 0.61	0.036 ± 0.002
	Bacteria	3217.257 ± 60.54	275.387 ± 11.61	0.086 ± 0.003
MA	Fungi	91.00 ± 4.08	3.38 ± 0.04	0.037 ± 0.001
	Bacteria	1483.25 ± 38.40	14.94 ± 1.53	0.014 ± 0.001
MB	Fungi	71.00 ± 6.73	1.61 ± 0.03	0.023 ± 0.002
	Bacteria	679.00 ± 23.16	9.46 ± 1.00	0.011 ± 0.001
GA	Fungi	336.50 ± 8.51	18.47 ± 0.10	0.015 ± 0.001
	Bacteria	2169.25 ± 18.40	92.41 ± 4.43	0.044 ± 0.001
GB	Fungi	388.00 ± 13.19	3.18 ± 0.05	0.008 ± 0.001
	Bacteria	1903.25 ± 9.72	17.357 ± 0.47	0.009 ± 0.001

*LA– Soil after *G. lucidum* cultivation; LB– Soil before *G. lucidum* cultivation.

MA– Wood segments after *G. lucidum* cultivation; MB– Wood segments before *G. lucidum* cultivation.

GA– *P. tabulaeformis* roots of the sites after *G. lucidum cultivation*; GB– *P. tabulaeformis* roots of the sites before *G. lucidum* cultivation.

### Fungal and bacterial community composition among soils, wood segments and tree roots

**Figure 1:**
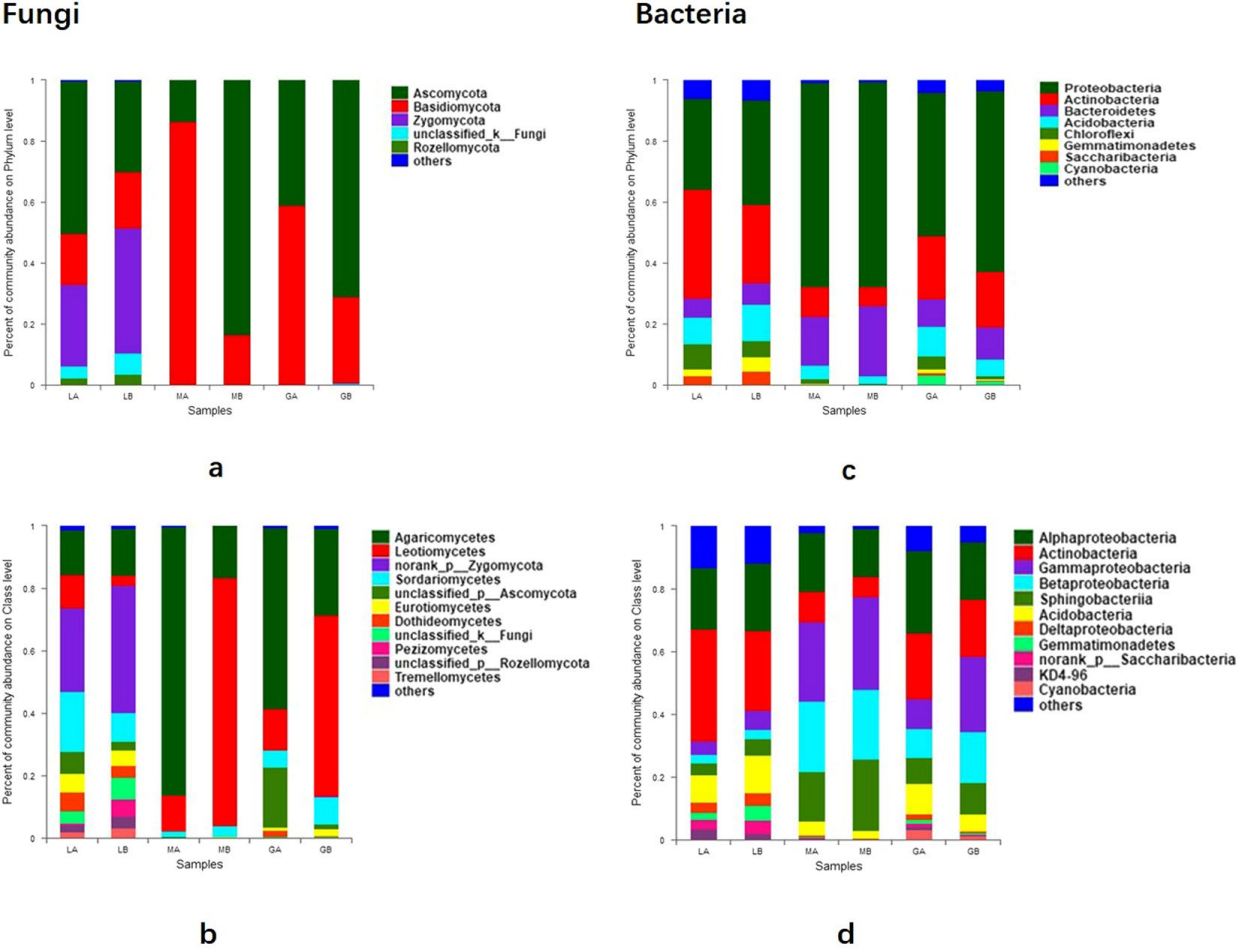
Fungal abundance in different samples: (**a**) at phylum level; (**b**) at class level; and bacteria abundance in different samples: (**c**) at phylum level; (**d**) at class level.

The sequences assigned to Fungi kingdom were classified into 7 fungal phyla. The relative abundances of phyla exceeding 1% in samples were shown in Fig. 1a. Totally, Ascomycota was the most abundant group (59.93%) followed by Basidiomycota (26.68%) and Zygomycota (6.83%). Others such as Glomeromycota weremuch less (<0.1%). Dominant fungi phyla changed after cultivation. For example, Ascomycota predominated in wood segments before cultivation, while, Basidiomycota took the place after cultivation. Abundance of Ascomycota and Basidiomycota showed significant differences between samples before and after *G. lucidum* cultivation (Supplementary Fig. S4A). Seven classes had a relative abundance of more than 1% (Fig. 1b), which include Agaricomycetes, Leotiomycetes, Sordariomycetes, Eurotiomycetes, Dothideomycetes, Pezizomycetes and Tremellomycetes. Agaricomycetes and Leotiomycetes showed significant differences between samples before and after cultivation (Supplementary Fig. S4B). Regarding the bacterial population, overall, Proteobacteria was most abundant, followed by Actinobacteria and Bacteroidetes. Phyla abundance were shown in Fig. 1c. Class Alphaproteobacteria, Actinobacteria and Gammaproteobacteria were most abundant (Fig. 1d). Abundance of 14 bacterial phyla including three phyla mentioned above and 14 classes showed significant differences before and after cultivation (Supplementary Fig. S3E,F).

**Figure 2:**
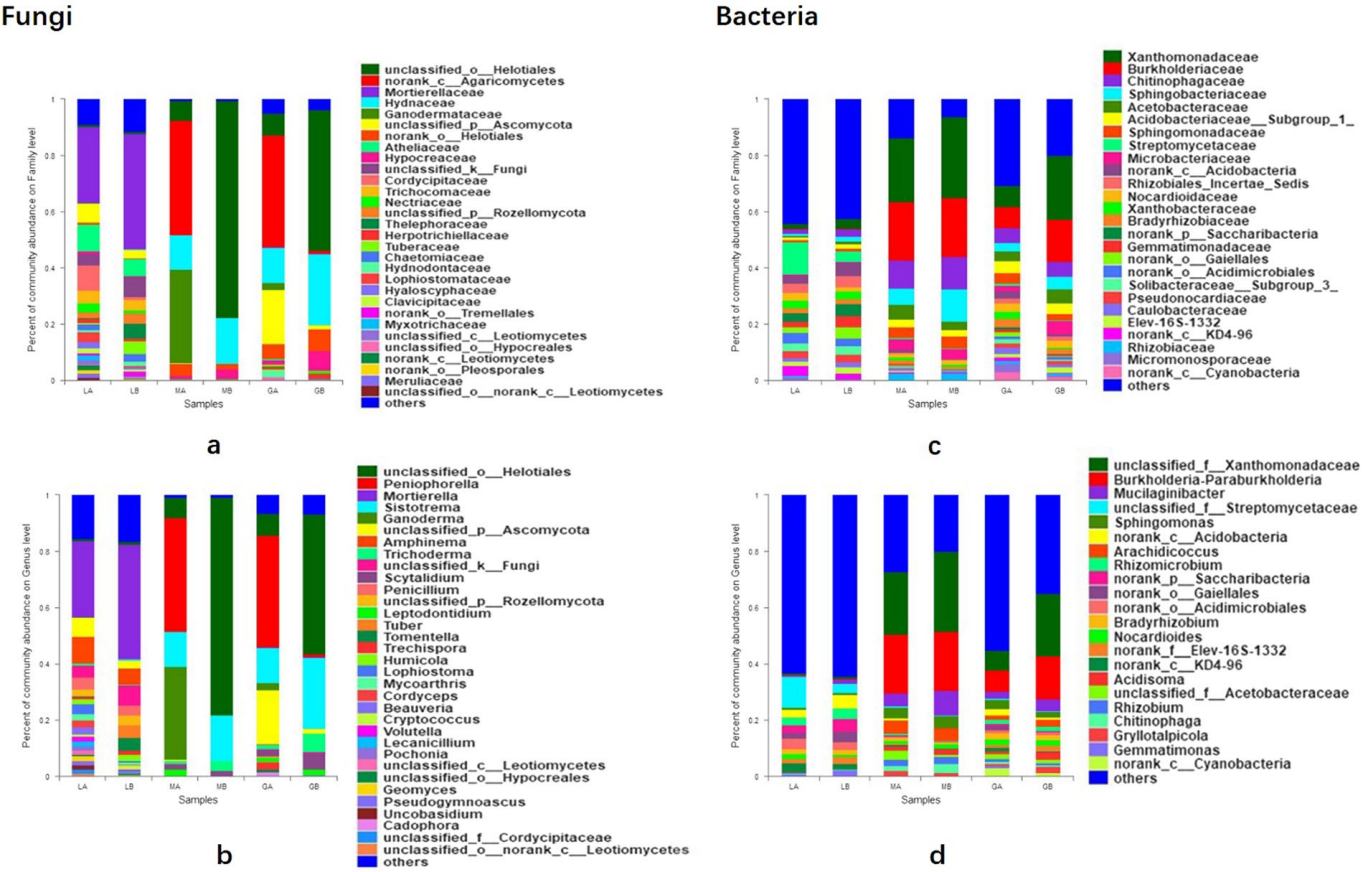
Fungal abundance in different samples: (**a**) at family level; (**b**) at genus level; bacterial abundance in different samples: (**c**) at family level; (**d**) at genus level.

At family level, relative abundance of 17 fungal and 18 bacterial groups exceeded 2% (Fig. 2a,c), such as eukarytic Mortierellacea (LA and LB), Ganodermatceae (MA and MB), Hydnaceae(GA and GB) and prokaryotic Streptomycetaceae (LA and LB; GA and GB), Sphingobacteriaceae (MA and MB) [Parentheses indicate great differences were shown between sampls].

As to genera, their relative abundance changed a lot after cultivation (Fig. 2b,d), such as eukarytic *Mortierella* (soil LA and LB), *Peniophorella* (MA and MB, GA and GB) and prokaryotic *Mucilaginibacter* (LA and LB), *Sphingomonas* (MA and MB, GA and GB). Many sequences can be just classified to genus or higher level. Relative abundance of 9 families and 11 genera (fungal), and 13 families and 7 genera (bacterial) including taxa mentioned above showed significant differences after cultivation (Supplementary Fig. S3C,D,G,H).

**Figure 3:**
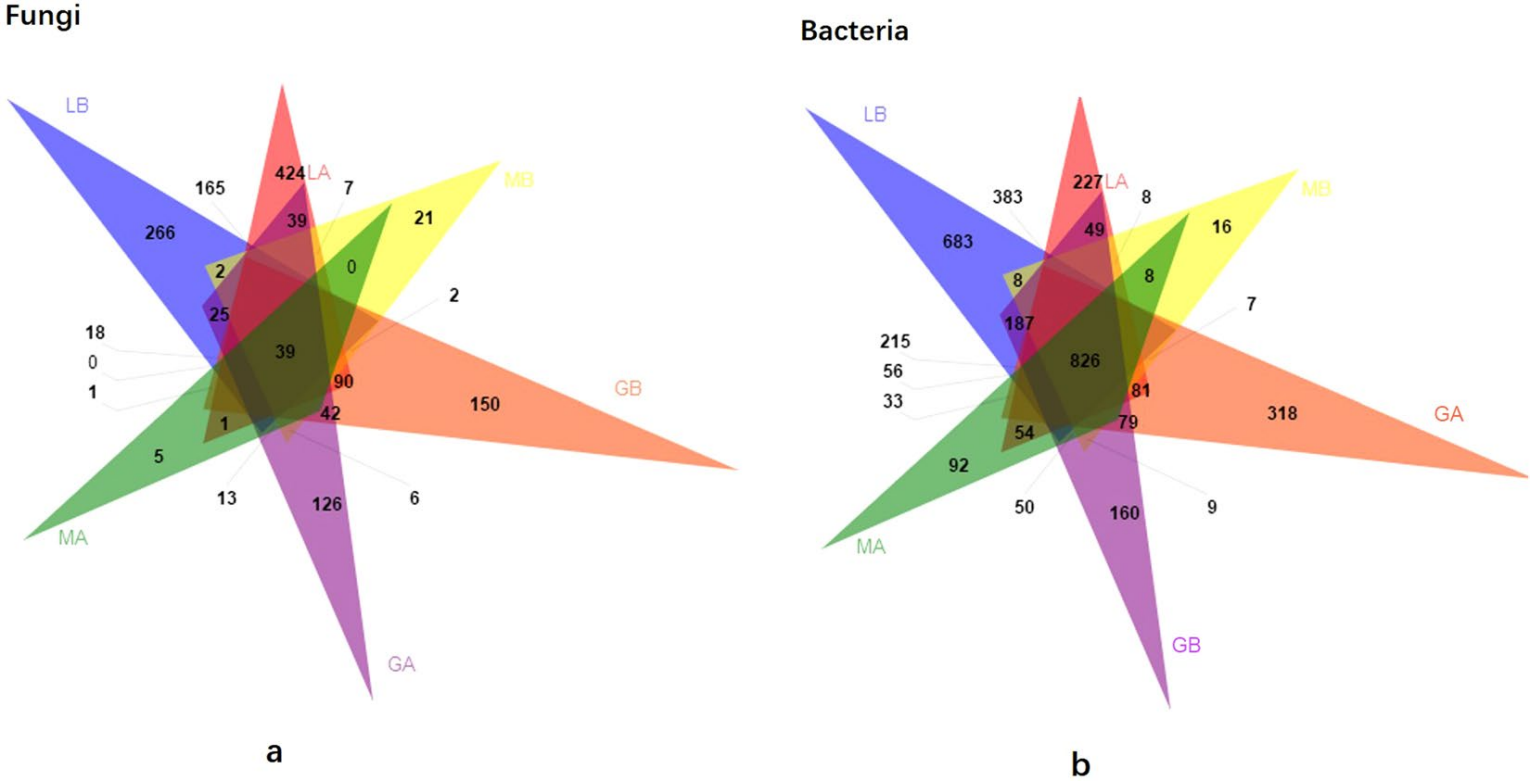
Venn diagram showing shared and unique (**a**) fungal; (**b**) bacterial OTUs in samples (soil, wood segments and tree roots before and after *G. lucidum* cultivation).

The common and unique OTUs of samples were illustrated in Fig. 3a,b. Fungi of LA had the most unique OTUs, while, Bacteria of LB had the most unique OTUs.

### The relationship between microbial community structure and *G. lucidum* cultivation and soil properties

**Figure 4:**
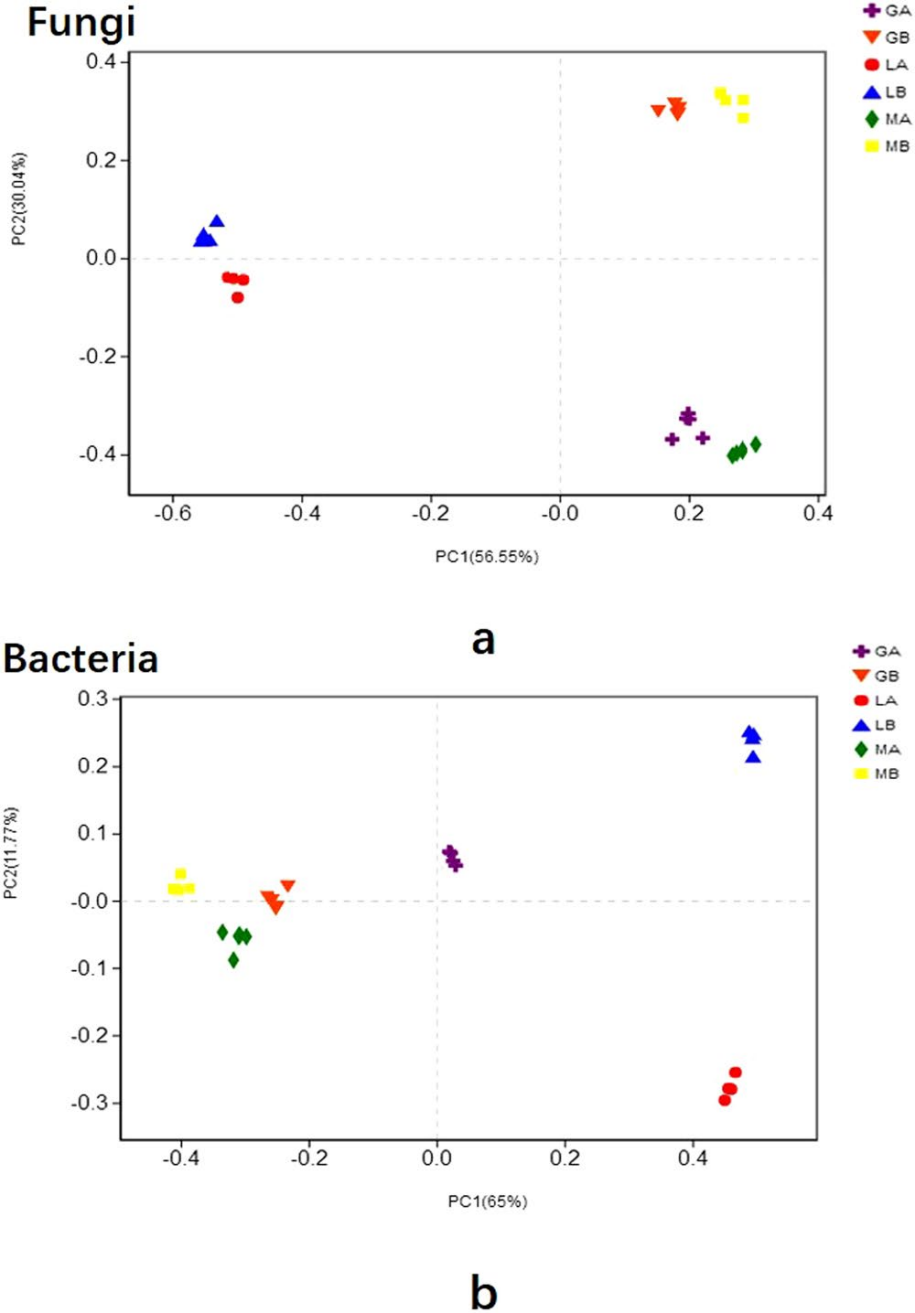
Principal coordinates analysis (PCoA) based on the relative abundance of microbial OTUs showing different microbial community structure: (**a**) fungi; (**b**) bacteria.

Principal Coordinate Analysis (PCoA) showed that soil microbial communities formed individual cluster before and after *G. lucidum* cultivation as well as in wood segments and tree roots (Fig. 4a,b). Subsequent PERMANOVA test confirmed the significant differences in community structures of all samples betwween two time stage (all *p* < 0.05).

**Figure 5:**
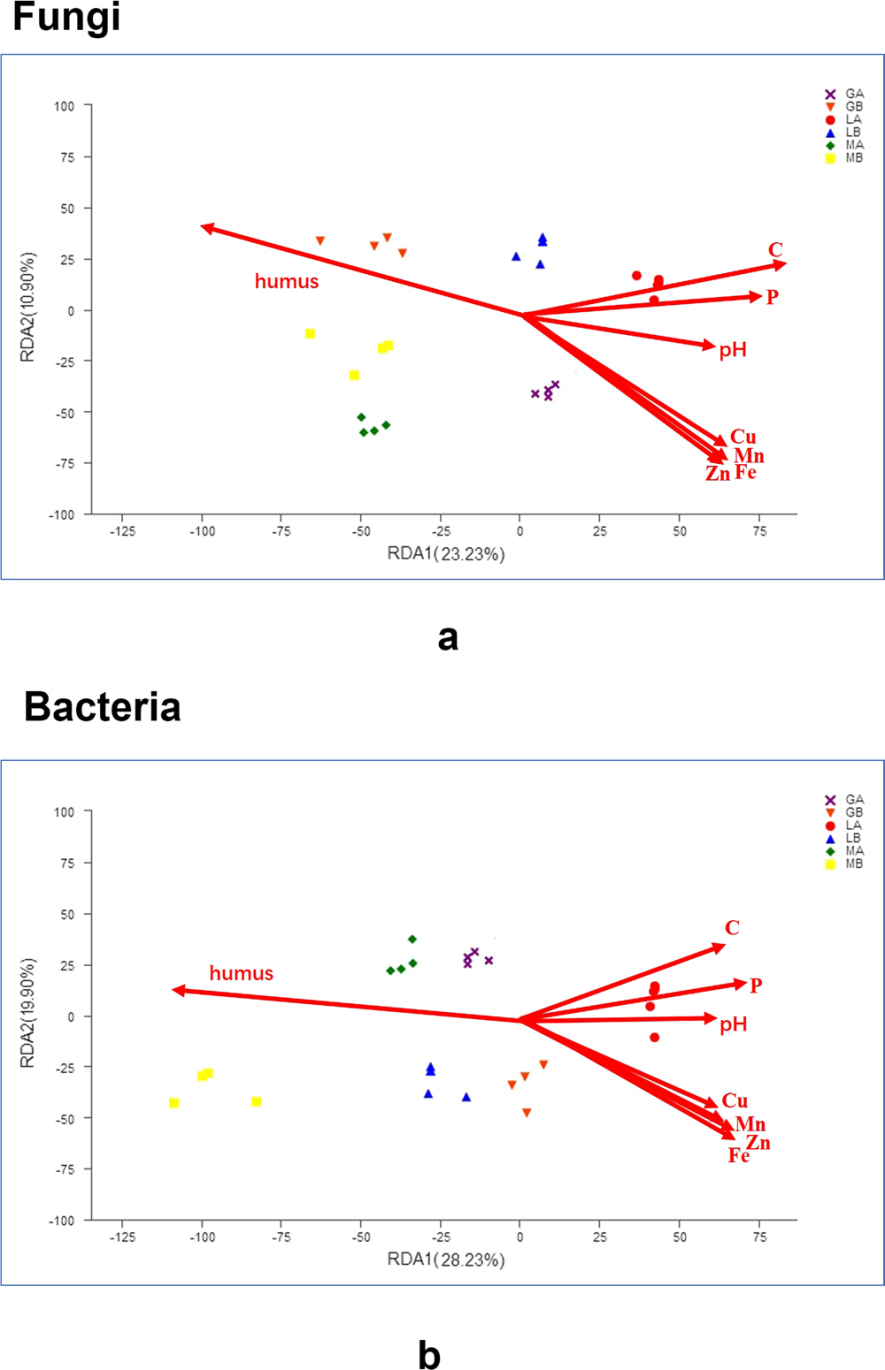
RDA plot showing the correlation between microbial community structure and soil properties: (**a**) fungi; (**b**) bacteria.

Soil property values are enclosed in Table 2. The values can be divided into three groups according to their change trend after cultivation: 1) reduced—humus; 2) almost stable—P, K, Na; 3) increased—pH, C, N, Fe, Zn, Mn, Cu. The RDA tests showed that the both fungal and bacterial community structure was significantly correlated with soil humus, pH, N, C, Fe, Cu, Zn and Mn (0.76 ≤ r^2^ ≤ 0.88; 0.001 ≤ *p* ≤ 0.002) (Fig. 5a,b). Trace elements (Fe, Zn, Mn, Cu) clustered closely, indicating similar effect degree, and showed same trend with C, N, pH (Fig. 5a,b). The tests results were somehow consistent with soil properties change trend.

**Table 2:** The soil properties before and after *G. lucidum* cultivation.

Soils properties	Before cultivation	After cultivation	*P* value
Soil C(g/kg)	31.4(±0.30)	48.5(±0.20)	0.001
Soil N(g/kg)	2.42(±0.05)	4.39(±0.02)	0.001
P (g/kg)	0.527(±0.004)	0.483(±0.02)	0.08
K (g/kg)	21.4(±0.2)	22.7(±0.20)	0.10
Na (g/kg)	14.0(±0.10)	14.7(±0.20)	0.10
Humus (g/kg)	48.5(±0.20)	30.4(±0.30)	0.001
Fe (mg/kg)	186(±1.20)	323(±1.50)	0.001
Zn (mg/kg)	23.4(±0.20)	61.7(±0.40)	0.001
Mn (mg/kg)	74.1(±0.50)	130(±0.60)	0.001
Cu (mg/kg)	1.88(±0.02)	2.47(±0.02)	0.001
pH	5.2(±0.05)	6.5(±0.04)	0.002

## Discussion

In our study, the microbial communities of soils, wood segments and tree roots before and after *G. lucidum* cultivation were mainly studied by Next-generation sequencing. 7 phyla, 28 classes, 95 orders, 206 families, 405 genera and 1,831 OTUs were identified in Fungi kingdom, 42 phyla, 93 classes, 198 orders, 401 families, 818 genera and 3,549 OTUs were determined in Bacteria, supplying a whole view for the uninvestigated microbial diversity and microbial succession during Ling Zhi cultivation. The study will also provide helpful information and potential use for *G. lucidum* cultivation and other edible and medicinal mushrooms cultivations to develop under-forest economic development.

Microbial richness, diversity and evenness indexes showing great differences after *G. lucidum* cultivation. The fungi cultivation can be taken as one kind of land use. Land use was considered as a main driving factor of bacteria for multiple land uses (arable, forest, grasslands)^25^. Previous studies also showed that microbial indexes could be influenced by land-use in many scale^26–28^. Some opposite change trends of fungi and bacteria in our research may indicate their interaction during Ling Zhi cultivation.

Microbial composition is influenced by land-use^25^. Featured fungal taxon was significantly related with distinct systems of land use^25^. Ascomycota predominated in wood segments and tree roots before Ling Zhi cultivation, while, Basidiomycota took the place after cultivation. Ascomycota showed an opposite trend in soils. Ascomycota and Basidiomycota showed significant differences between samples before and after *G. lucidum* cultivation. Changes of fungal community of soil were also reported especially distinctive among trophic groups^29^. Bacterial Proteobacteria and Actinobacteria were most abundant and also differed greatly in our study. The most represented phyla Proteobacteria and Actinobacteria were also found in different land use soils along European transect^25^. The dominance of the two phyla is in agreement with other studies of soill^30,31^. Class Agaricomycetes and Leotiomycetes showed significant differences between samples before and after cultivation. Agaricomycetes has about 21,000 recorded mushroom-forming species as pathogens, decayers and mutualists of terrestrial and aquatic habitats^32^. Their abundance was gradually declined with longer length with grazing exclusion in soil layers^33^. Leotiomycetes is one of the largest nonlichen-forming ascomycetous groups, include varieties of ecology of saprobes, plant endophytes, plant pathogens, fungi-parasites, and insect and other animal associates^34^. Actinobacteria is one of the largest groups of Bacteria, very significant in the biodegradation and recycling of organic matter^35^. Abundance changes exhibit the effects of Ling Zhi cultivation.

Abundance of dominant taxa including *Mortierella, Ganoderma, Hydnaceae* etc. changed after cultivation. *Mortierella* of Mortierellacea have been reported to produce arachidonic acid^36^ and single cell oil (SCO)^37^*. Ganoderma* of Ganodermatceae showed significant difference of MA and MB. This is not difficult to understand since Ling Zhi grew directly on wood segments. Ling Zhi cultivation obviouslyincrease *Ganoderma* abundance. Hydnaceae includes many wood−decay fungi, and is vital in carbon and nutrient recycling in forest ecosystems^38^. Abundance of some prokaryotic taxa also altered remarkably. Streptomycetaceae belongs to Actinobacteria, members of it can produce a wide array of clinically important bioactivemolecules^39^, and have been found in plants roots, and affect tree growth^40^. Its abundance showed great changes after cultivation, especially in soils and tree roots. Sphingobacteriaceae, were found existed in all of soil samples of different days after paraquat use^41^, among various strains of *Tyrophagus putrescentiae*^42^. *Mucilaginibacter* members exist in many soil samples^43,44^. The genus was hypothesized to play an important role in processing carbon^44^. *Sphingomonas* has been reported having abilities of degradation of polyphenols and biodegradation of tributyl phosphate (TBP)^45,46^. The ecological roles and functions of the taxa in our study merits further research.

Structure of microbial communities differed significantly before and after *G. lucidum* cultivation. Each of the sample (soils, wood segments and tree roots) in our study harbored a different microbial assemblage. Continuous monoculture of watermelon for twenty years significantly change the soil microbes, adding to diversity of bacteria, while, reducing fungal diversity^8^. Microbes are critical in farmland ecosystems, particularly in improving soil fertility. Significant changes were also found in microbial communities caused by mudflat reclamation under rice cultivation^47^. The fungal community composition of soil showed notable differences among the first, the secondand the third rotation plantation during consecutive monoculture in *Casuarina equisetifolia* plantations^48^. The results in our study show consistency with these findings. However, wood preservatives with copper can delay wood breaking down and altered fungi but not bacteria composition of soil^49^. More studies are needed in the future.

Soil property values also changed much after cultivation, humus reduced, pH, C, N, and trace elements (Fe, Zn, Mn, Cu) increased, indicating cultivation put profound effect on soil properties. Microbial community structure was also obiviously connected with soil properties, exhibiting soil may affect the associated microbial community. Importance of plants and soil on belowground microbial diversity has been investigated in the last decades^50^. Studies at local to continental-level showed that soil properties are major driver effectors of microbial communities of soil, and also profound with plant communities and land use^25^. Close mutual relationship was made between plants and soils. Plants can call for their rhizosphere microbes from the soil pool^51^. The microbial communities structure of pear trees also obviously correlated with properties of the growth soil microbial communities of root and rhizosphere are much more similar^52^. In our study, soil, *G. lucidum* cultivation, and forest tree roots may have a very complicated interaction. Wood segments, soils and tree roots can provide nutrients and affect Ling Zhi growth, and Ling Zhi growth can produce many beneficial molecular and compounds^1–3^, influencing wood segments, soils, and tree roots and tree growth. A recent study showed that planting with peanut monocropping and crop rotation greatly affected the rhizosphere microbial assembly of the peanut^53^. It also has reported that *G. lucidum* can produce a lot of chemicals like *Ganoderma lucidum* Polysaccharide, Ganodenic acid etc., having the ability of anti-tumor, anti-aging and detoxification^1–3^. Recent studies have found that *G. lucidum* polysaccharides produce anti-inflammatory, anti-obesity and anti-diabetes effects by modulating gut microbiota composition in mice^54,55^. These may help explain soil and microbial differences after Ling Zhi cultivation in some degree. The microbial group in the study might be isolated and used to improve soil quality in the future. The results could be drawn for other mushrooms cultivation under forests. The study will also provide information for sustainable management of soil and make better understandings of the influences of cultivations on microbes.

## Conclusions

Microbial compositions of the samples (soils, wood segments and tree roots) in our study were not same. Structure of microbial communities differed significantly before and after *G. lucidum* cultivation. Soil property values also changed much after cultivation. Both composition of fungal and bacterial community had a remarkble correlation with soil humus, pH, N, C, Fe, Cu, Zn and Mn contents. Our results indicate that *G. lucidum* cultivation might have important effects on the associated composition of microbial community, soil properties and tree growth. The microbial roles of ecology and functions will merit be studied in the future.

## Supplementary information

Supplementary Figures.
