# Supplementary material for: Ganoderma lucidum cultivation affect microbial community structure of soil, wood segments and tree roots
Source: Sci Rep. 2020 Feb 26;10:3435. doi: 10.1038/s41598-020-60362-2 (PMC7044327; doi:10.1038/s41598-020-60362-2)
Supplement: Supplementary file 1 — Supplementary Figures. [file 41598_2020_60362_MOESM1_ESM.pdf]

Ganoderma lucidum cultivation affect microbial community structure of soil, wood segments and tree roots

Fei Ren[1,3*], Yuguang Zhang[2], Hai Yu[1], Yong An Zhang[1]*

[1] Forestry experiment center in north China, Chinese Academy of Forestry; feiren@caf.ac.cn, zhangyab@caf.ac.cn

[2] Research Institute of Forest Ecology, Environment and Protection, Chinese Academy of Forestry; yugzhang@sina.com.cn

[3] Academy of National Food and Strategic Reserves Administration

Supplementary Material

Supplementary Figure S1. Samples, including soils, wood segments and *Pinus tabulaeformis* roots.

Supplementary Figure S2. Statistically significant differences in the fungal species richness (A), diversity (B) and evenness (C) of the samples, bacterial species richness (E), diversity (F) and evenness (G) of the samples *$0.01 < p \leqslant 0.05$，**$0.001 < p \leqslant 0.01$，***$p \leqslant 0.001$.

Supplementary Figure S3. Rarefaction curves of all the samples. (A) fungi, (B) bacteria. All the samples are saturated for further study.

Supplementary Figure S4. Significantly different taxa among samples. Fungi: (A) at phylum level, (B) at class level, (C) at family level, (D) at genus level; Bacteria: Bacteria: (E) at phylum level, (F) at class level, (G) at family level, (H) at genus level.

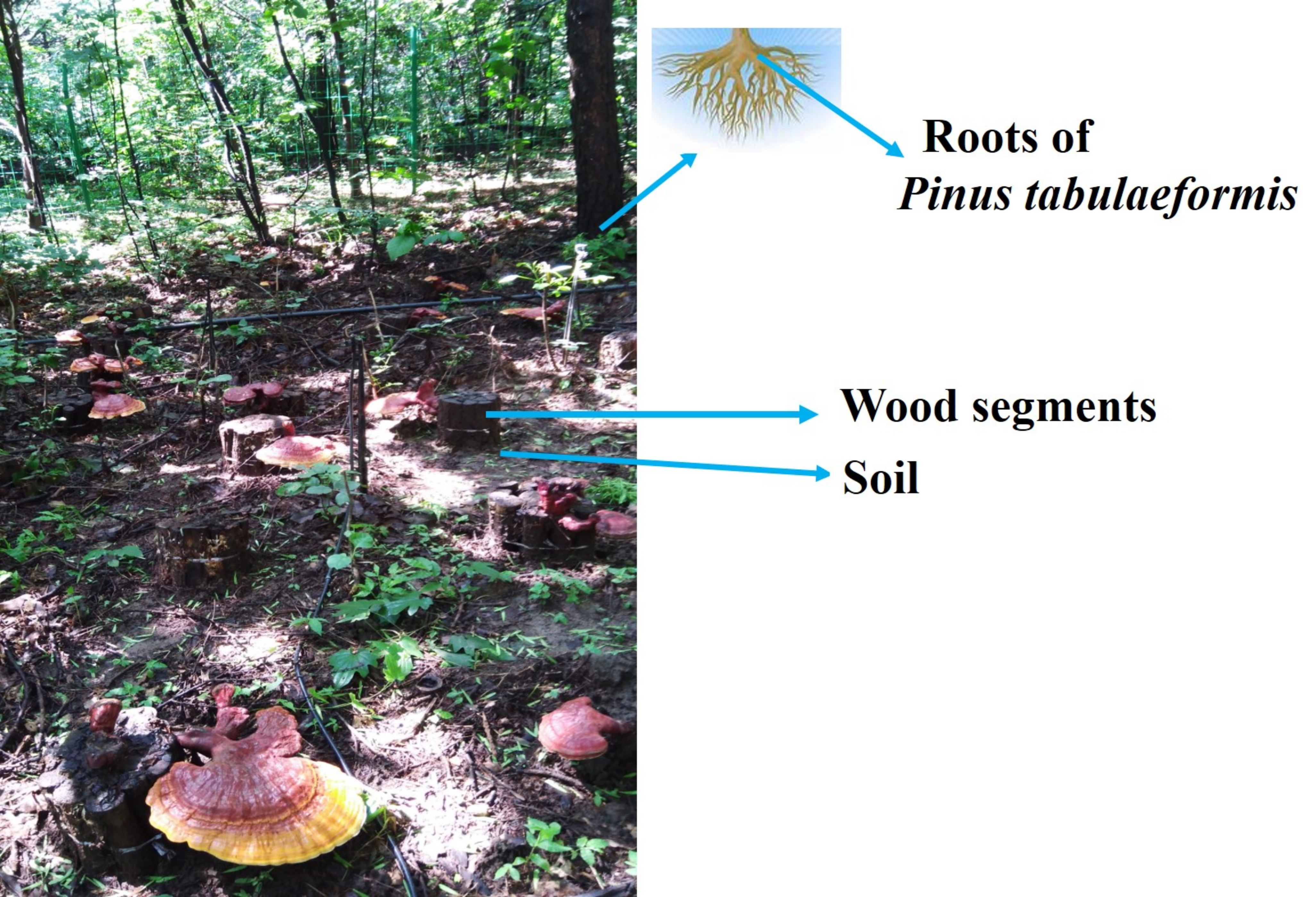
Roots of
Pinus tabulaeformis
Wood segments
Soil

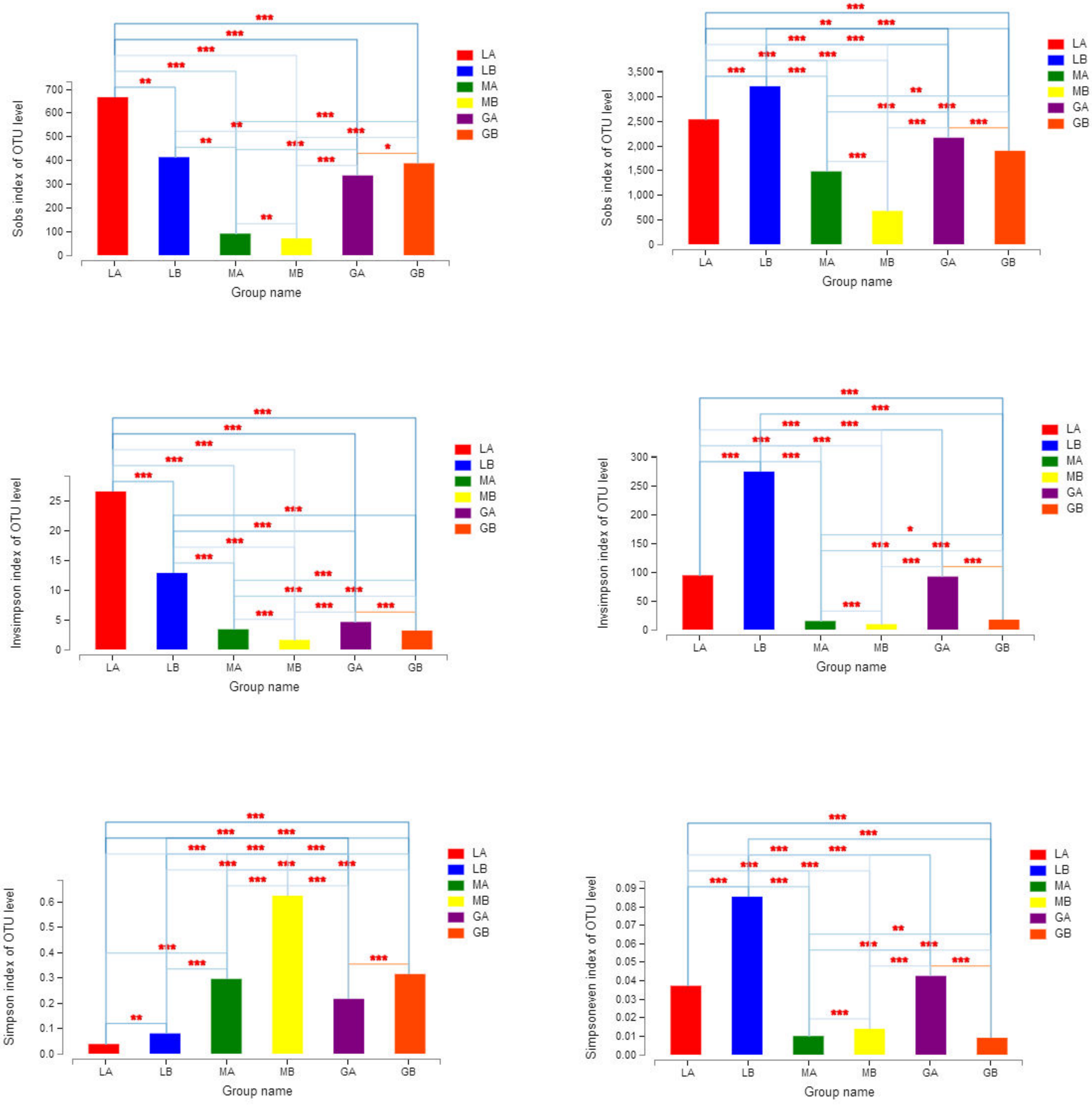
Sobs index of OTU level
Invsimpson index of OTU level
Simpson index of OTU level
Simpsoneven index of OTU level
Group name
LA
LB
MA
MB
GA
GB

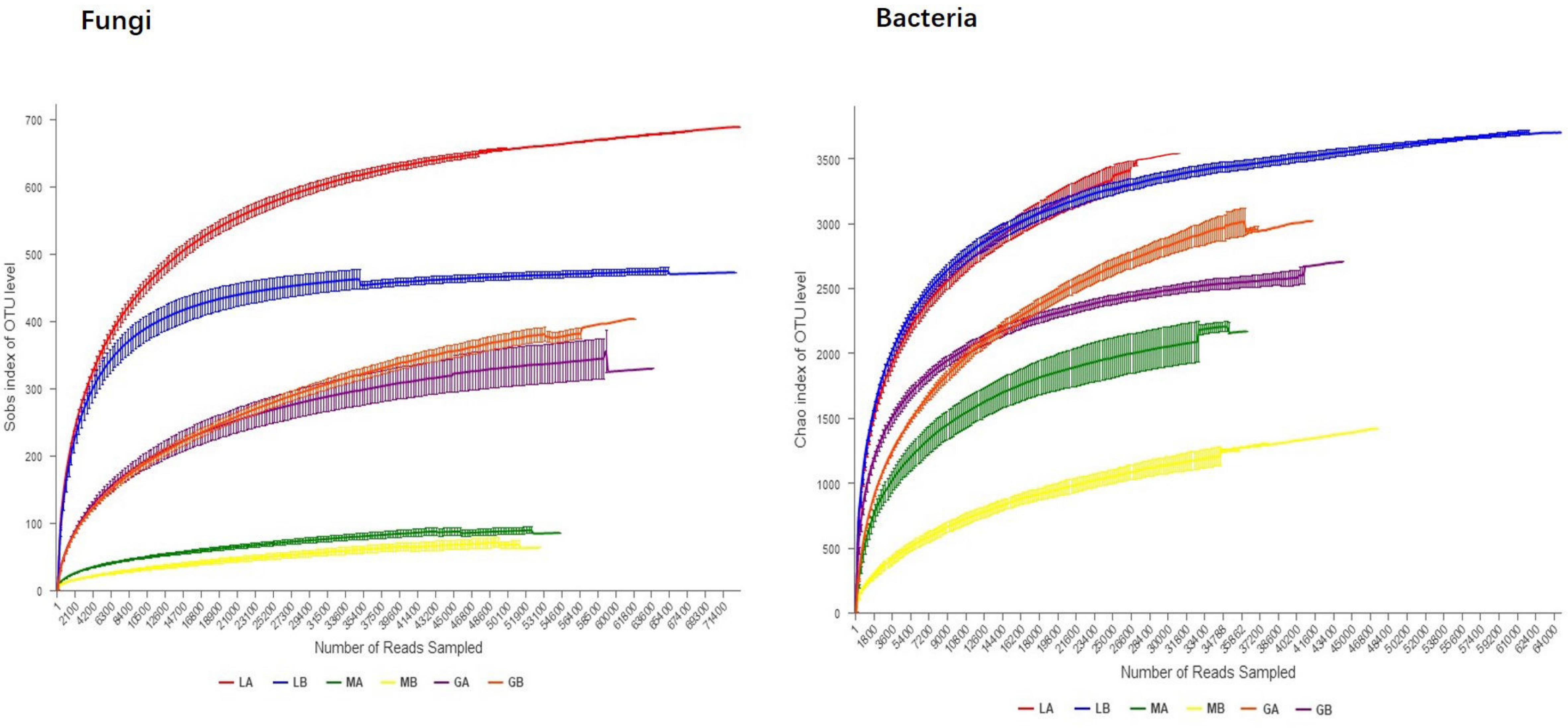
Fungi
Sobs index of OTU level
0
100
200
300
400
500
600
700
Number of Reads Sampled
LA
LB
MA
MB
GA
GB
Bacteria
Chao index of OTU level
0
500
1000
1500
2000
2500
3000
3500
Number of Reads Sampled
LA
LB
MA
MB
GA
GB

A

B

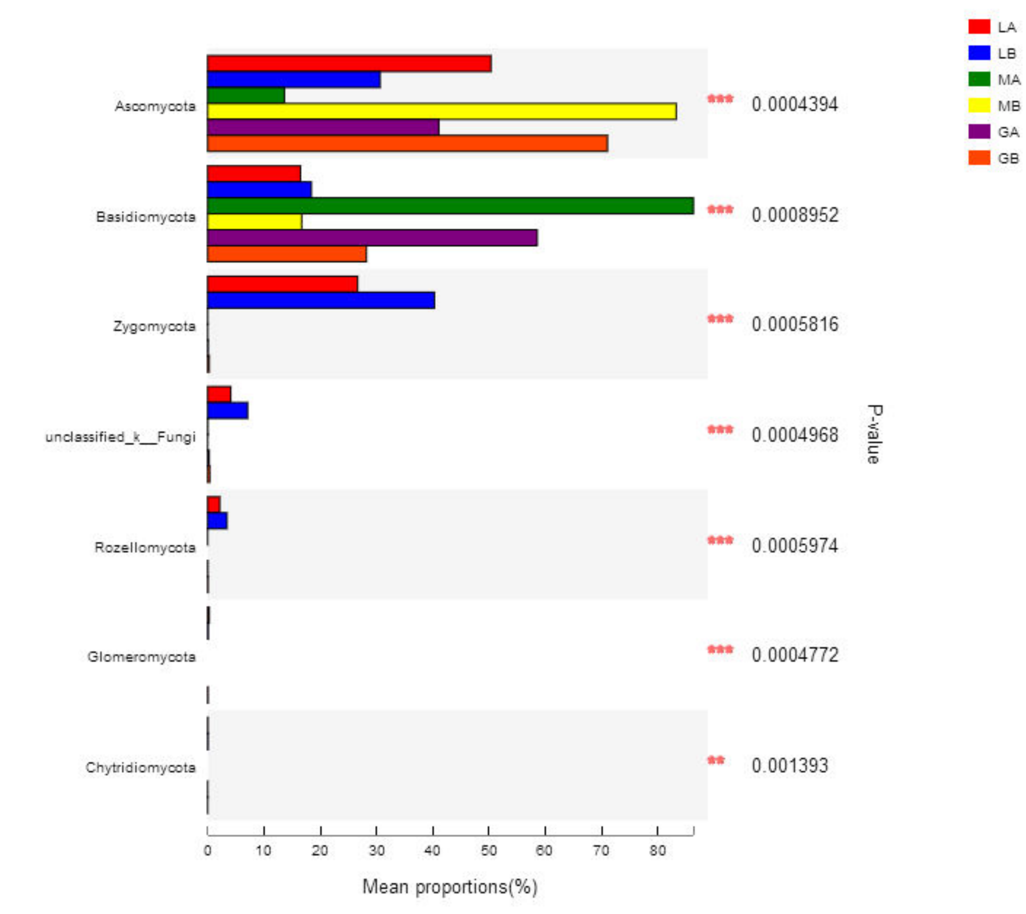
Ascomycota
Basidiomycota
Zygomycota
unclassified_k__Fungi
Rozellomycota
Glomeromycota
Chytridiomycota
Mean proportions(%)
P-value
LA
LB
MA
MB
GA
GB

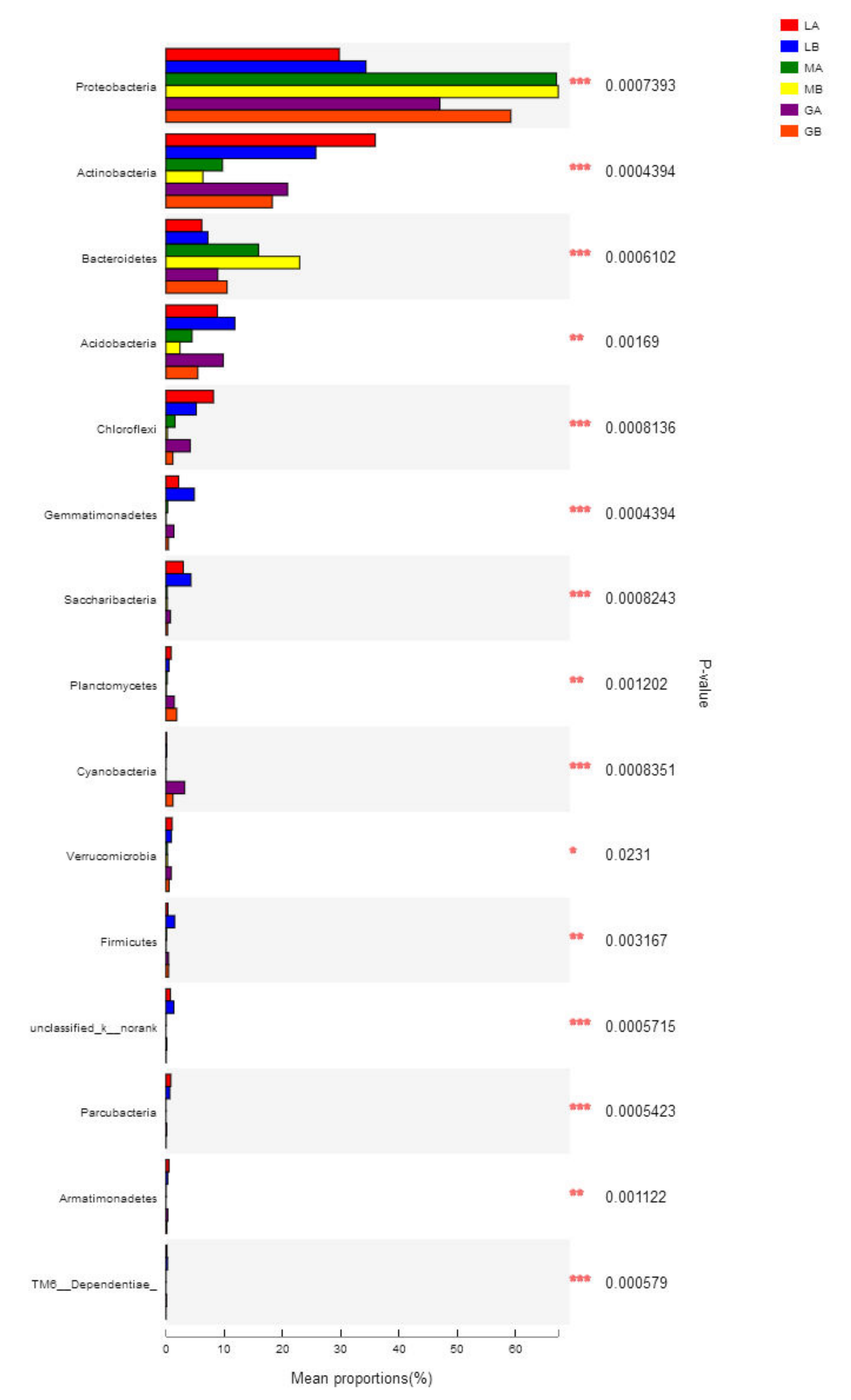
Proteobacteria
Actinobacteria
Bacteroidetes
Acidobacteria
Chloroflexi
Gemmatimonadetes
Saccharibacteria
Planctomycetes
Cyanobacteria
Verrucomicrobia
Firmicutes
unclassified_k__norank
Parcubacteria
Armatimonadetes
TM6__Dependentiae_
Mean proportions(%)
P-value
LA
LB
MA
MB
GA
GB

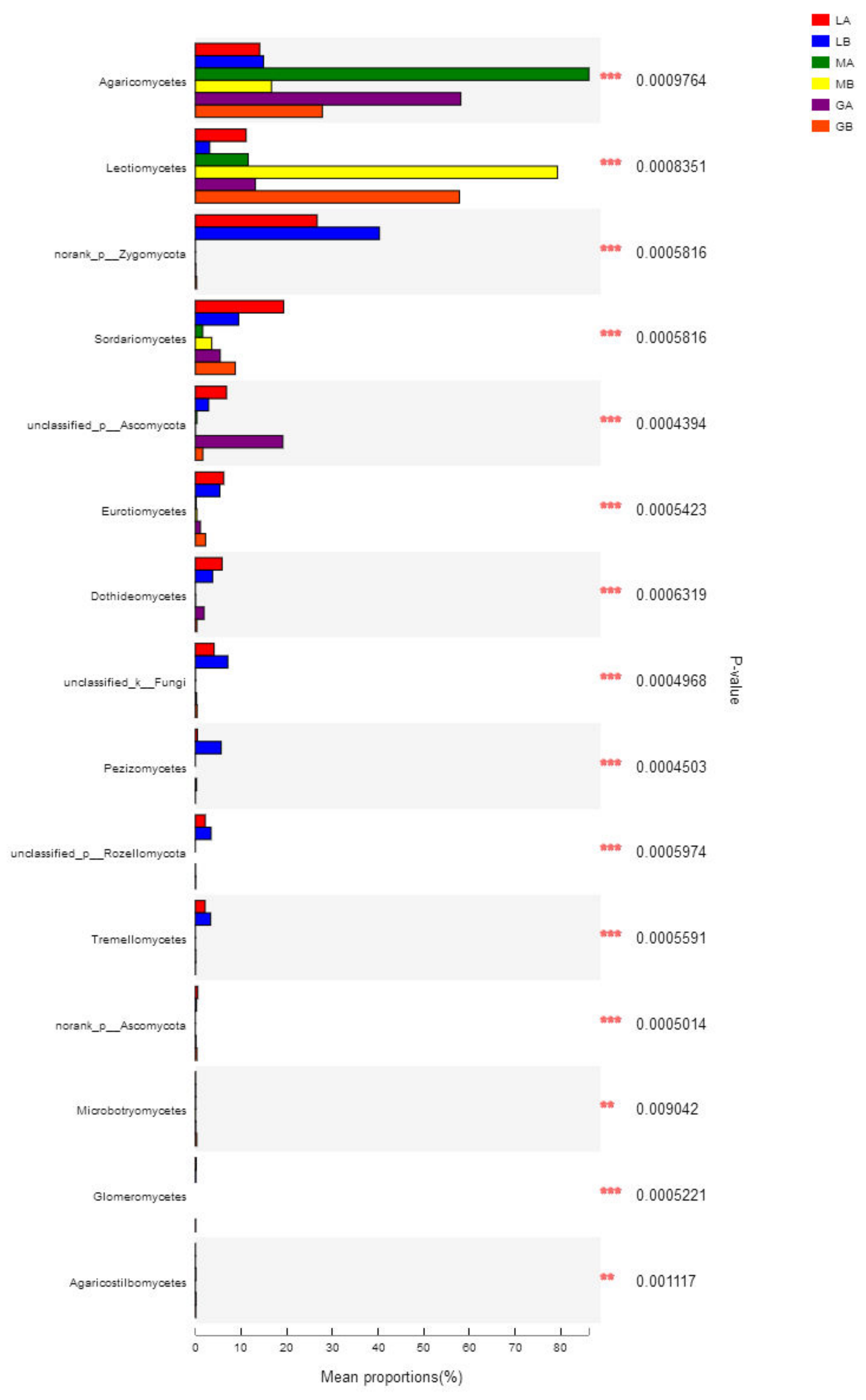
Agaricomycetes
Leotiomycetes
norank_p__Zygomycota
Sordariomycetes
unclassified_p__Ascomycota
Eurotiomycetes
Dothideomycetes
unclassified_k__Fungi
Pezizomycetes
unclassified_p__Rozellomycota
Tremellomycetes
norank_p__Ascomycota
Microbotryomycetes
Glomeromycetes
Agaricostilbomycetes
Mean proportions(%)
P-value
LA
LB
MA
MB
GA
GB

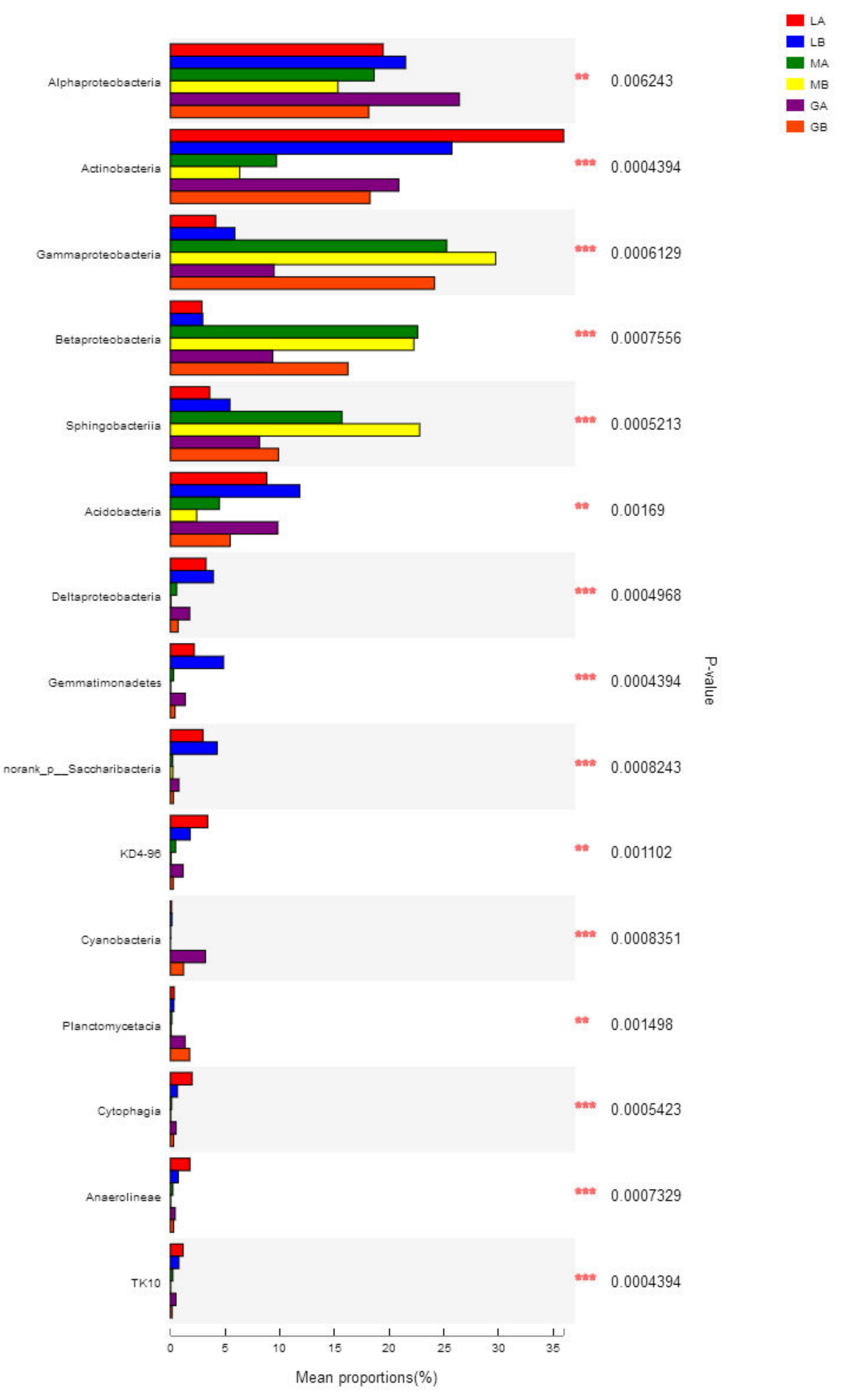
Alphaproteobacteria
Actinobacteria
Gammaproteobacteria
Betaproteobacteria
Sphingobacteriia
Acidobacteria
Deltaproteobacteria
Gemmatimonadetes
norank_p__Saccharibacteria
KD4-96
Cyanobacteria
Planctomycetacia
Cytophagia
Anaerolineae
TK10
Mean proportions(%)
P-value
LA
LB
MA
MB
GA
GB

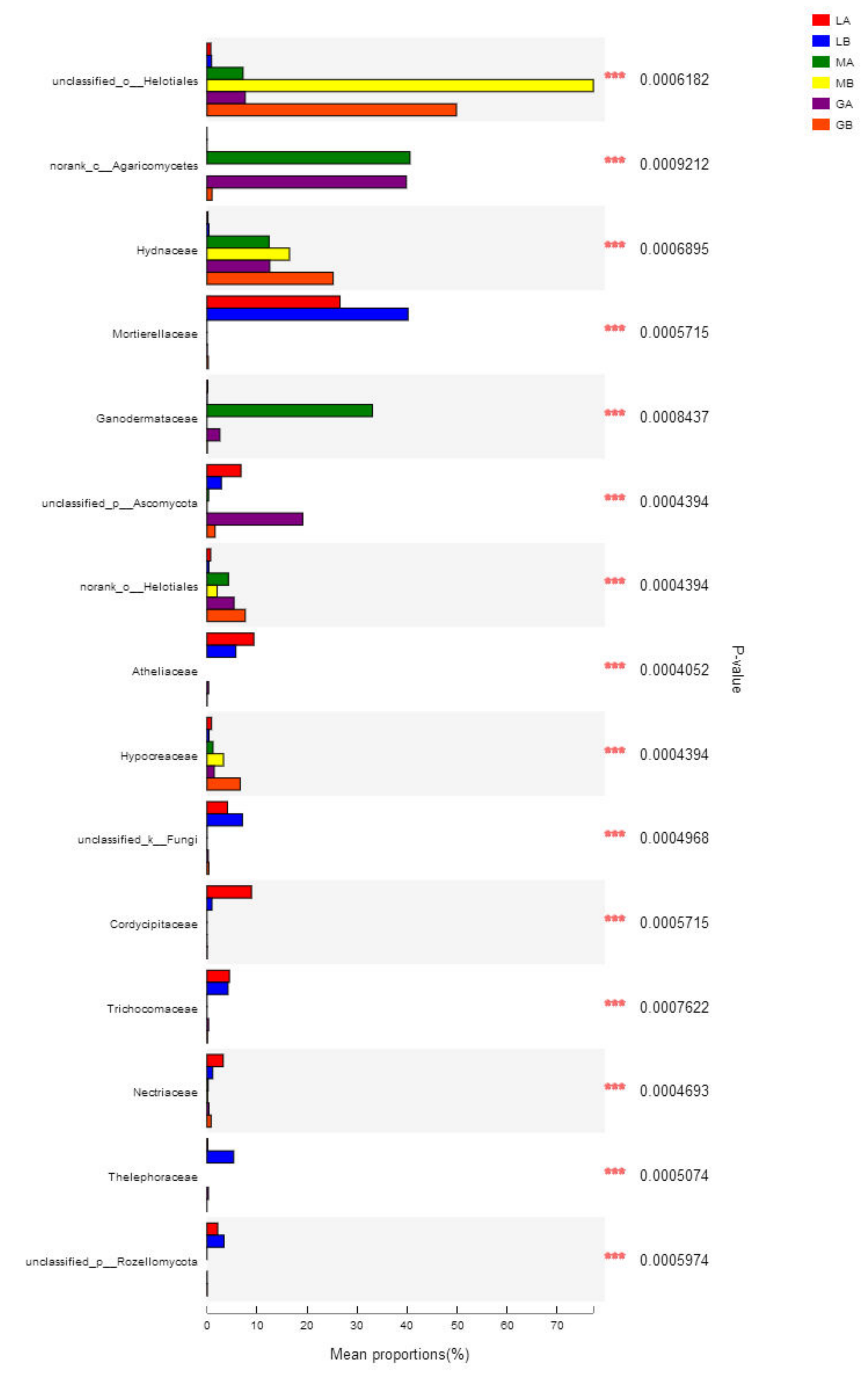
unclassified_o__Helotiales
norank_o__Agaricomycetes
Hydnaceae
Mortierellaceae
Ganodermataceae
unclassified_p__Ascomycota
norank_o__Helotiales
Atheliaceae
Hypocreaceae
unclassified_k__Fungi
Cordycipitaceae
Trichocomaceae
Nectriaceae
Thelephoraceae
unclassified_p__Rozellomycota
Mean proportions(%)
P-value
LA
LB
MA
MB
GA
GB

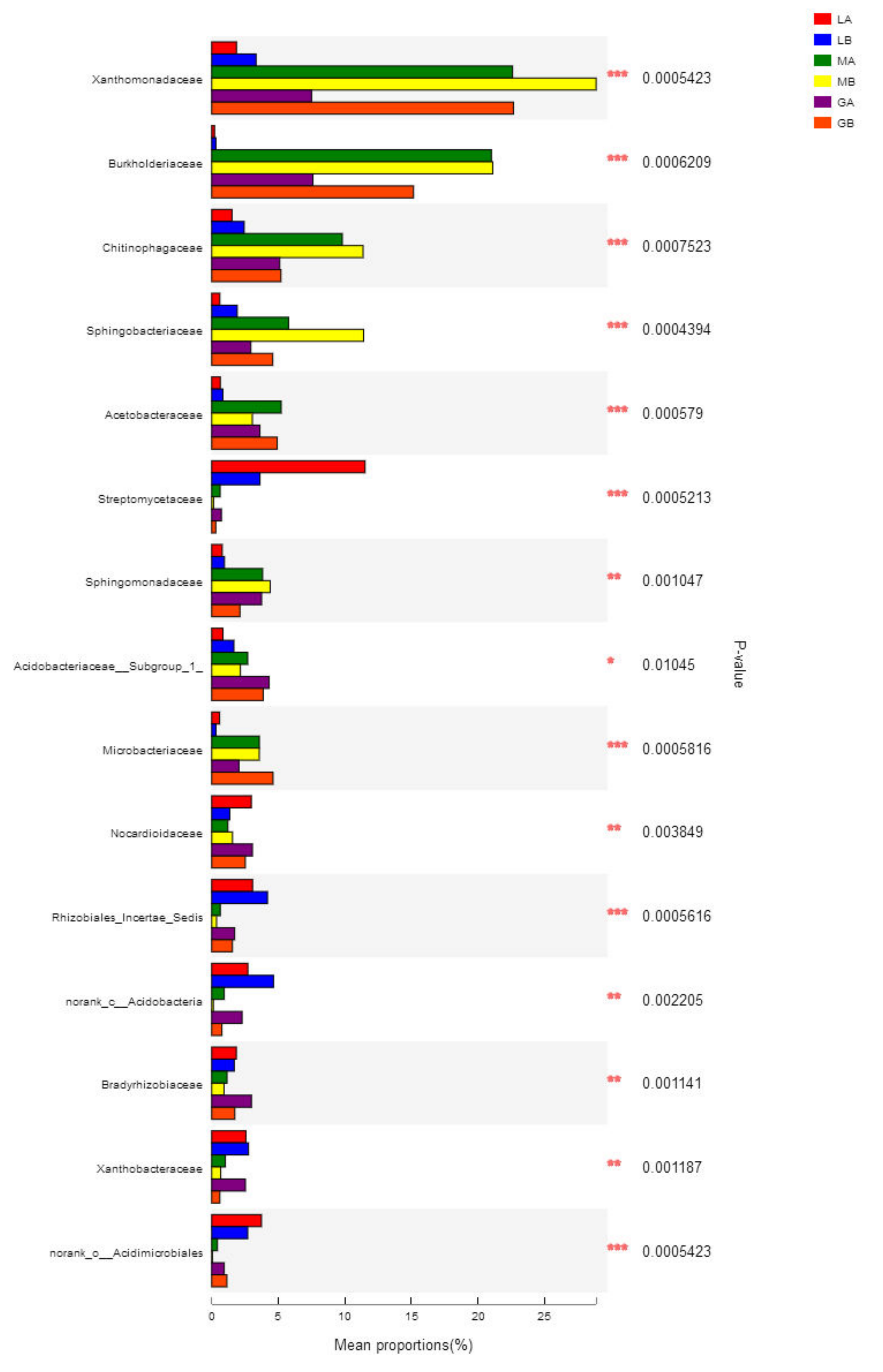
Xanthomonadaceae
Burkholderiaceae
Chitinophagaceae
Sphingobacteriaceae
Acetobacteraceae
Streptomycetaceae
Sphingomonadaceae
Acidobacteriaceae__Subgroup_1_
Microbacteriaceae
Nocardioidaceae
Rhizobiales_Incertae_Sedis
norank_c__Acidobacteria
Bradyrhizobiaceae
Xanthobacteraceae
norank_o__Acidimicrobiales
Mean proportions(%)
P-value
LA
LB
MA
MB
GA
GB

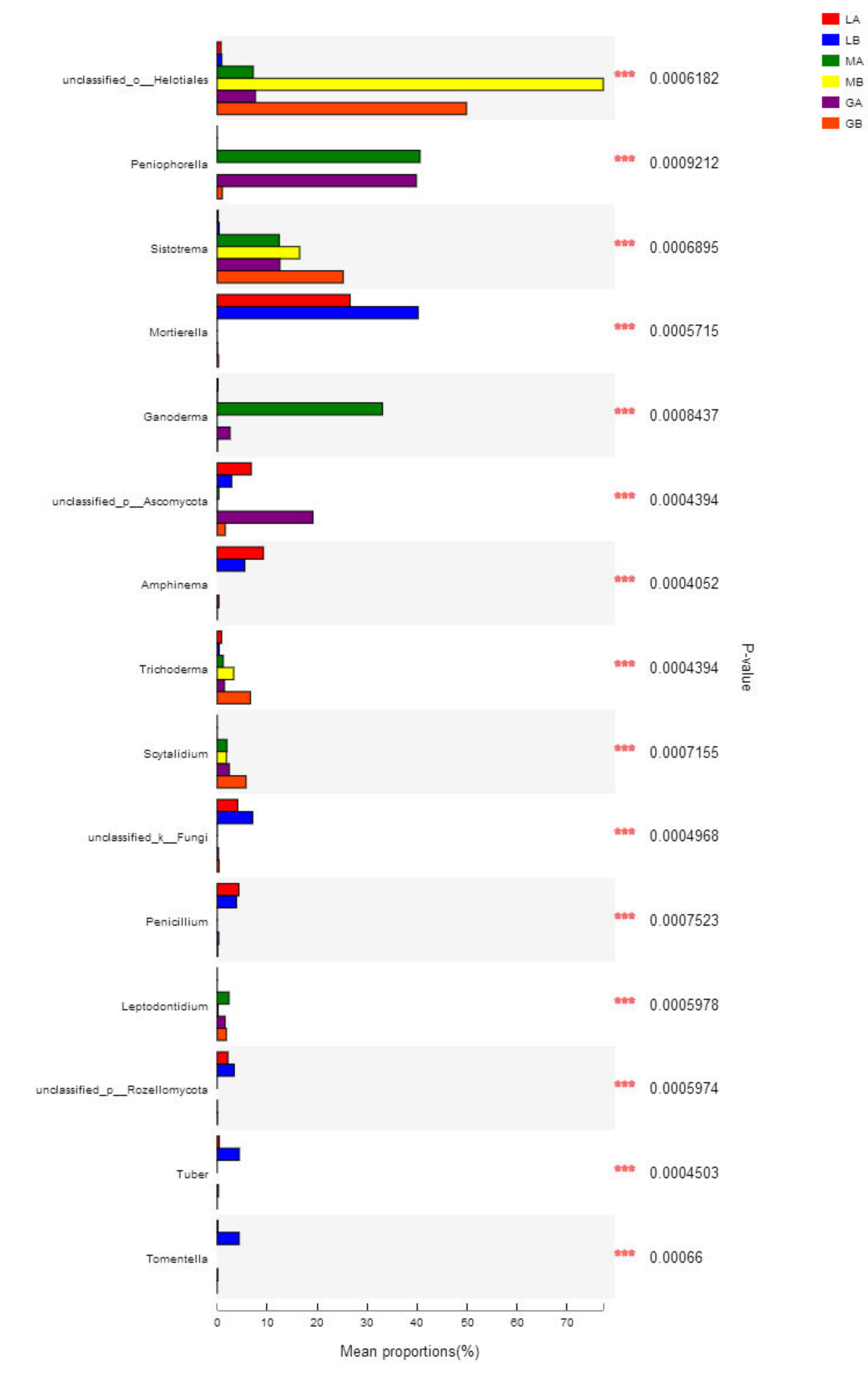
unclassified_o__Helotiales
Peniophorella
Sistotrema
Mortierella
Ganoderma
unclassified_p__Ascomycota
Amphinema
Trichoderma
Scytalidium
unclassified_k__Fungi
Penicillium
Leptodontidium
unclassified_p__Rozellomycota
Tuber
Tomentella
Mean proportions(%)
P-value
LA
LB
MA
MB
GA
GB

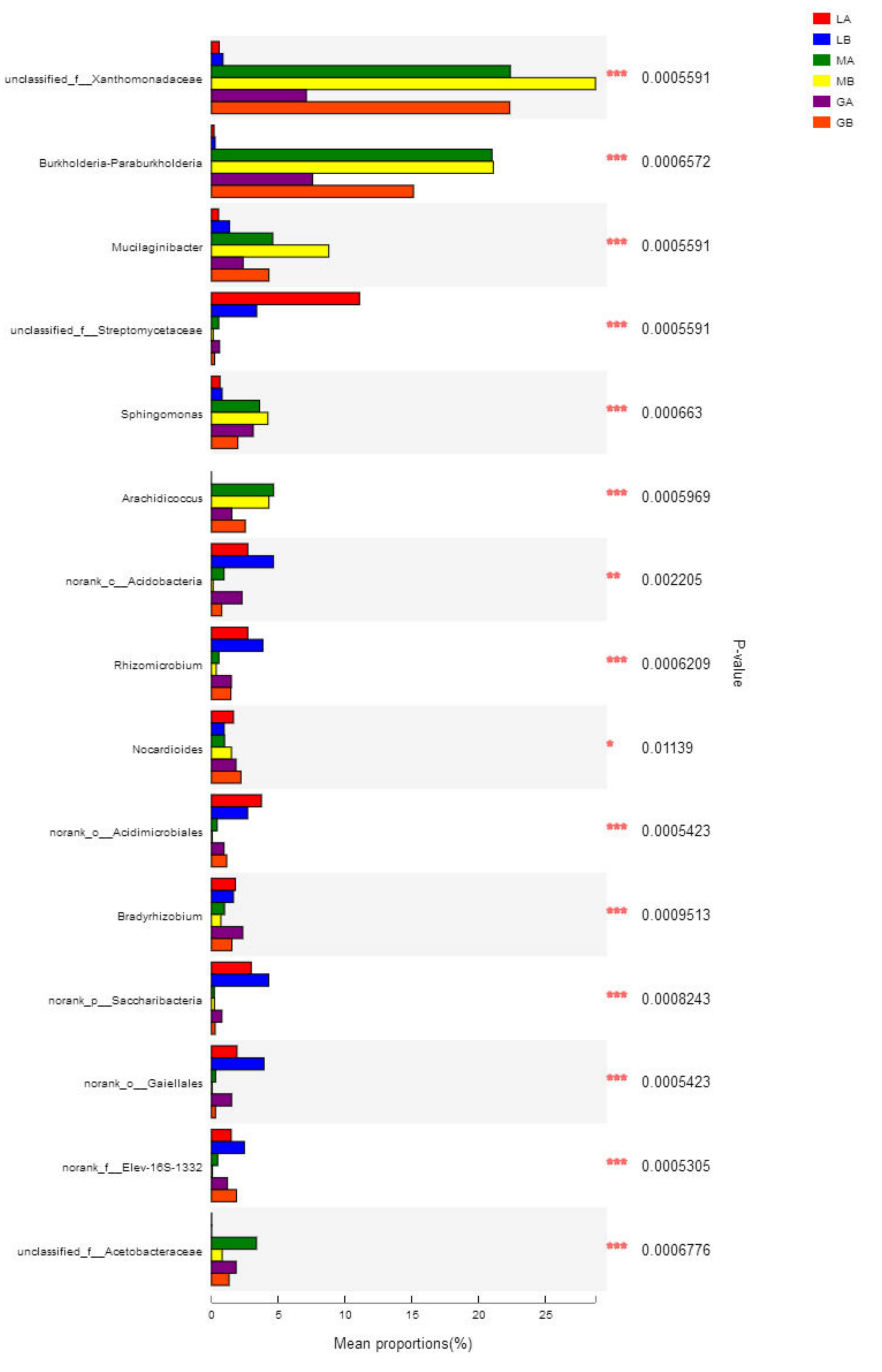
unclassified_f__Xanthomonadaceae
Burkholderia-Paraburkholderia
Mucilaginibacter
unclassified_f__Streptomycetaceae
Sphingomonas
Acidibacter
norank_c__Acidobacteria
Rhizomicrobium
Nocardioides
norank_o__Acidimicrobiales
Bradyrhizobium
norank_p__Saccharibacteria
norank_o__Gaiellales
norank_f__Elev-16S-1332
unclassified_f__Acetobacteraceae
Mean proportions(%)
P-value
LA
LB
MA
MB
GA
GB
